# Comparison of Chikungunya Virus-Induced Disease Progression and Pathogenesis in Type-I Interferon Receptor-Deficient Mice (A129) and Two Wild-Type (129Sv/Ev and C57BL/6) Mouse Strains

**DOI:** 10.3390/v16101534

**Published:** 2024-09-27

**Authors:** Victoria A. Graham, Linda Easterbrook, Emma Rayner, Stephen Findlay-Wilson, Lucy Flett, Emma Kennedy, Susan Fotheringham, Sarah Kempster, Neil Almond, Stuart Dowall

**Affiliations:** 1 UK Health Security Agency (UKHSA), Porton Down, Salisbury SP4 0JG, Wiltshire, UK; victoria.graham@ukhsa.gov.uk (V.A.G.); linda.easterbrook@ukhsa.gov.uk (L.E.); emma.rayner@ukhsa.gov.uk (E.R.); stephen.findlay-wilson@ukhsa.gov.uk (S.F.-W.); lucy.flett@ukhsa.gov.uk (L.F.); emma.kennedy@ukhsa.gov.uk (E.K.); susan.fotheringham@ukhsa.gov.uk (S.F.); 2 Medicines and Healthcare Products Regulatory Agency (MHRA), Blanche Ln, South Mimms, Potters Bar EN6 3QG, Hertfordshire, UK; sarah.kempster@mhra.gov.uk (S.K.); neil.almond@mhra.gov.uk (N.A.)

**Keywords:** Chikungunya, mouse, model, preclinical, pathogenesis

## Abstract

Chikungunya virus (CHIKV) is a mosquito-borne alphavirus causing a debilitating febrile illness with rheumatic disease symptoms of arthralgia and arthritis. Since its spread outside of Africa in 2005, it continues to cause outbreaks and disseminates into new territories. Intervention strategies are urgently required, including vaccination and antiviral approaches. To test efficacy, the use of small animal models is required. Two mouse strains, A129, with a deficiency in their type-I interferon (IFN) receptor, and C57BL/6 are widely used. A direct comparison of these strains alongside the wild-type parental strain of the A129 mice, 129Sv/Ev, was undertaken to assess clinical disease progression, viral loads in key tissues, histological changes and levels of sera biomarkers. Our results confirm the severe disease course in A129 mice which was not observed in the parental 129Sv/Ev strain. Of the two wild-type strains, viral loads were higher in 129Sv/Ev mice compared to C57BL/6 counterparts. Our results have established these models and parameters for the future testing of vaccines and antiviral approaches.

## 1. Introduction

Chikungunya virus (CHIKV) is an alphavirus within the *Togaviridae* family [1]. CHIKV was first isolated in 1952 from a human patient in Tanzania [2] with the virus being named after the Makonde word for “that which bends you up”, in recognition of the severe joint pains associated with the disease [3].

For decades only local and occasional outbreaks were documented, until 2004 when CHIKV emerged at the coast of Kenya [4]. CHIKV is primarily transmitted by *Aedes aegypti* and *Aedes albopictus* mosquitoes, with a key mutation in the E1 gene (A226V) providing the virus with a gain-of-fitness adaptation enabling more efficient transmission by the latter species [5]. This likely contributed to the spread and magnitude of the outbreaks outside of mainland Africa in the Indian Ocean islands in 2005–2006, and subsequently across several countries in Southeast Asia [6,7]. In 2013, a CHIKV outbreak occurred on the Caribbean island of St. Martin [8] and thereafter, in 2014, spread to Brazil and across the American continent, resulting in more than 1.2 million cases in a single year [9]. *Aedes albopictus* are present in several areas of Europe [10], thus expanding the potential for the emergence of CHIKV into new geographical regions. This has been exemplified by the first autochthonous cases reported in Italy during 2007 [11] and France in 2010 [12].

CHIKV is listed as a priority pathogen by the UK Vaccine Network (UKVN) [13] and the Coalition for Epidemic Preparedness Innovations (CEPI) [14]; therefore, a healthy pipeline of vaccine candidates exist. One vaccine produced by Valneva has demonstrated seroprotective titres in a Phase 3 clinical trial [15], and has subsequently been approved by the US Food & Drug Administration (FDA) and European Medicines Agency (EMA). In addition, several antiviral compounds have been identified, but few have been assessed in animal models which is required to confirm their mode of action and effect [16]. The C57BL/6 mouse is wildly used in research due to substantial genetic homology with humans [17]. This strain has been used across multiple studies with CHIKV infection [18,19,20,21,22,23,24,25]. Similarly, A129 mice with a deficiency in their type-I interferon receptor (and similar knock-out strains) are widely susceptible to viral disease, including CHIKV [20,22,23,26]. The wild-type parental strain, 129Sv/Ev, has been less used. This study therefore compared these three mouse strains after challenge with CHIKV, delivered subcutaneously near the foot to resemble natural infection via mosquito biting and feeding. 

## 2. Materials and Methods

### 2.1. Virus

Chikungunya virus (strain LR 2006-OPY1) was kindly gifted from the Commissariat à l’Energie Atomique et aux Energies Alternatives (CEA) for use in this study. The virus originated from a French patient returning from La Reunion Island and passaged 3 times in Vero cells before a stock was produced on BHK-21 cells. Virus stock was titred on Vero cells to be 1.8 × 10^8^ plaque-forming units/mL.

### 2.2. Animals

All experimental protocols with animals were undertaken according to the United Kingdom (UK) Animals (Scientific Procedures) Act 1986, with studies conducted under the authority of a UK Home Office approved project licence. The experimental protocols were approved by ethical review at UKHSA by the Animal Welfare and Ethical Review Body (AWERB; Approval Code: PPL P82D9CB4B). Female mice aged 5–8 weeks were obtained from UK Home Office approval suppliers: Marshall BioResources (strain A129 and 129Sv/Ev) and Envigo (C57BL/6). Mice were randomly allocated and housed in groups of 5–6 with food and water available *ad libitum* alongside regular environmental enrichment provided within cages. During and after challenge with CHIKV, all procedures, housing and husbandry took place inside a flexible film isolator housed within a Containment Level 3 facility. Prior to the start of the study, humane clinical endpoints were set which consisted of 20% weight loss compared to baseline; inactivity/immobility; neurological signs; or based on the advice of severe disease from the Named Animal Care and Welfare Officer (NACWO).

### 2.3. Study Design

**Figure 1:**
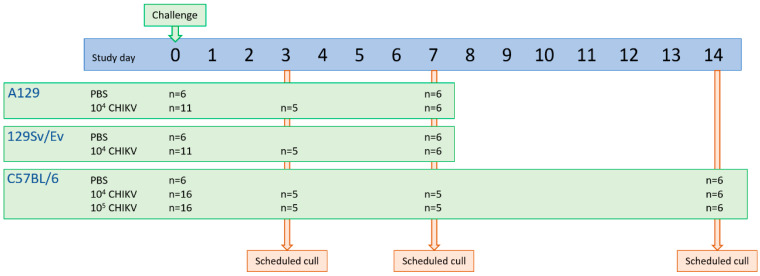
Schematic overview of study design assessing CHIKV infection in three mouse strains.

To assess differences between the different mouse strains, according to the study groups, 5 mice were scheduled to be sampled at each timepoint based on power calculations (Wilcoxon and Mann–Whitney test, effect size 2.5, 95% significance and equal group allocation; G*Power 3.1.9.4). For survival analysis, 6 mice were assigned to each group based on a one-tailed Fisher’s exact test (significance threshold of 0.05, 6 animals would give >80% power of demonstrating the difference to the control; G*Power 3.1.9.4). At day 3 and 7 post-challenge, groups were scheduled for cull to assess local responses between groups. The scheduled end of the study was day 7 for A129 mice and their 129Sv/Ev parental strain, and 14 days for the C57BL/6 strain. An overview of the study design is shown in Figure 1.

### 2.4. Challenge, Monitoring and Sampling

Mice were anaesthetised with isoflurane before being inoculated via the subcutaneous route with 40 μL of CHIKV or PBS control into the cranio-dorsal aspect of each distal hindlimb, just proximal to the tarsal joint. CHIKV was diluted with PBS to a ensure a concentration of either 10^4^ or 10^5^ pfu in the 80 μL inoculum distributed across the two limbs.

Body weight and temperature were monitored daily at the same time of day, the latter via an indwelling temperature chip (identiCHIP). Clinical and behavioural parameters were assessed and scored at least twice a day, with the frequency increasing to four times a day when observations considered to be moderate in severity were observed. Each observation was assigned a numerical value [1, eyes shut; 2, ruffled fur; 3, abnormal posture (hunched or arched), lethargy; 5, laboured breathing]; these were subsequently summed to derive a total cumulative score at each monitoring timepoint. 

At necropsy, the right distal hindlimb below the stifle and samples of spleen and brain were placed into a PreCellys tube containing ceramic beads and stored at −80 °C for viral RNA assessment. The left distal hindlimb below the stifle and remainder of the spleen and brain were placed into 10% neutral-buffered formalin (NBF) for pathological examination. Blood was collected via cardiac puncture, with 100 μL added to animal RNAprotect blood tubes (Qiagen, Manchester, UK) and stored at −80 °C for viral RNA measurement. The remainder was placed into serum separation tubes (SST; Becton Dickinson, Wokingham, UK) with sera processed and stored at −80 °C for measurement of analytes by Luminex assay.

### 2.5. Viral RNA Measurement

Tissue samples for viral RNA analysis were weighed, resuspended in 1.5 mL PBS and homogenised using a PreCellys 24 homogeniser (Stretton Scientific, Alfreton, UK). Two hundred µL of tissue homogenate or blood was transferred to 600 µL RLT buffer (Qiagen, Manchester, UK) plus beta-mercaptoethanol and after at least 10 min, mixed with an equal volume of 70% ethanol. Tissues were further homogenised through a QIAshredder (Qiagen, Manchester, UK) at 16,000× *g* for 2 min and RNA extracted by KingFisher Flex automatic extraction using the BioSprint 96 one-for-all veterinary kit (Indical, Leipzig, Germany) as per manufacturer’s instructions. RNA was eluted in 100 µL AVE buffer (Qiagen, Manchester, UK). 

Samples were analysed by qRT-PCR using an assay that detects and quantifies CHIKV-specific amplicons via the one-step RT-PCR method [27]. The sensitivity of this method was determined using a series of 10-fold dilutions of in vitro transcribed RNA, generated from a cloned cDNA copy of the amplicon (pCH127), allowing readouts to be standardised to genome copies. 

### 2.6. Histopathological Studies

The tissue samples fixed in 10% NBF were processed as follows: the hindlimb tarsal joint was transected sagittally and decalcified in ‘Osteosoft’ (Sigma Aldrich, Gillingham, UK) for 14 days. Together with sections of the spleen and brain, these were processed routinely into paraffin wax. Sections were cut to 4 µm and stained with haematoxylin and eosin (H&E). In addition, an in situ hybridisation technique (‘RNAscope^®^’) was used to detect the presence of CHIKV RNA. Briefly, tissues were pre-treated with hydrogen peroxide for 10 min (room temperature), followed by target retrieval for 15 min (98–101 °C) and protease plus for 30 min (40 °C) (Advanced Cell Diagnostics, Abingdon, UK). Tissues were then incubated with a CHIKV-specific probe (Catalogue no. 479508, Advanced Cell Diagnostics) for 2 h at 40 °C. Amplification of the signal was carried out following the RNAscope protocol using the RNAscope 2.5 HD Detection kit—Red (Advanced Cell Diagnostics). 

Stained slides were scanned digitally using a ‘Hamamatsu S360’ digital slide scanner and examined using ‘ndp.view2’ software (v2.8.24) on a 4K digital monitor by a qualified pathologist who was blinded to the animal group details to minimise bias. Microscopic changes noted within the skeletal muscle, skin and subcutis were evaluated using a subjective scoring system that graded them as minimal, mild, moderate, or marked (for details of scoring methodology for skeletal muscle, skin and subcutis of the hindlimb samples, see Appendix A). Due to challenges encountered during microtomy, the joint tissues, including bone, were not present consistently in the sections and were therefore not scored. For the evaluation of the degree of staining for viral RNA, the following scoring system was used: 0 = no positive staining; 1 = minimal; 2 = mild; 3 = moderate; and 4 = abundant staining.

### 2.7. Luminex Analysis

A 32-plex mouse cytokine/chemokine panel was used consisting of granulocyte colony-stimulating factor (G-CSF), granulocyte-macrophage colony-stimulating factor (GM-CSF), eotaxin, interferon-gamma (IFNγ), interleukin(IL)-1a, IL-1β, IL-2, IL-3, IL-4, IL-5, IL-6, IL-7, IL-9, IL-10, IL-12(p40), IL-12(p70), IL-13, IL-15, IL-17, IFNγ-inducible protein 10 (IP-10), keratinocyte chemoattractant (KC), leukaemia inhibitory factor (LIF), lipopolysaccharide-induced CXC (LIX), macrophage colony-stimulating factor (M-CSF), macrophage inhibitory protein (MIP)-1α, MIP-1β, MIP-2, monocyte chemotactic protein 1 (MCP-1), monocyte induced by IFNγ (MIG), regulated upon activation, normal T cell expressed and presumably secreted (RANTES), tumor necrosis factor alpha (TNFα) and vascular endothelial growth factor (VEG-F) (Millipore, Watford, UK). The assay was performed according to the manufacturer’s instructions. A standard preparation was diluted with assay buffer to produce a range covering concentrations of 10,000, 2000, 400, 80, 16 and 3.2 pg/mL. Within the Containment Level 3 (CL3) laboratory—required for handling samples from CHIKV-infected animals—25 μL of standard and quality control preparations or 25 µL sera were added to the relevant wells followed by 25 μL of serum matrix and 25 μL of premixed beads supplied with the kit. Plates were incubated at 4 °C overnight before being washed twice with 200 μL/well washing buffer using a handheld magnet. Following the wash steps, 25 μL of detector antibodies were added to all wells and the plate incubated for 1 h at room temperature. Next, 25 μL of streptavidin-phycoerythrin solution was added to each well and the plate was incubated for a further 30 min without any washing steps. After completion of staining, the microbeads were washed twice with wash buffer. To remove plates from the CL3 laboratory for analysis, beads were treated with formalin, previously shown to be amendable to this assay [28,29]. Beads were resuspended with 100 μL/well of 10% formalin solution made by dilution of 100% formalin (40% *w*/*v* formaldehyde solution) (Scientific Laboratory Supplies, Nottingham, UK) 1:9 *v*/*v* with phosphate buffered saline solution (Thermo Fisher, Loughborough, UK). Plates were fumigated with formaldehyde vapour overnight at room temperature for 16 h with the lids left ajar to allow vapour to reach all surfaces. Following fumigation, plates were placed into a sealed bag and removed from the CL3 laboratory.

Plates were washed twice with wash buffer, and once with sheath fluid in a Containment Level 2 laboratory to remove formalin solution before being resuspended in 150 µL of sheath fluid. Results were acquired on a Luminex MAGPIX instrument using Exponent software (v4.2.1324.1; Invitrogen, Paisley, UK). At least 50 events per region were collected and median fluorescence intensity (MFI) was measured. MFI values were converted to concentrations using results from the standard curve preparations. 

### 2.8. Statistical Analysis

Statistical analyses were performed using MiniTab, v.16.2.2 (Minitab Inc, State College, PA, USA). A non-parametric Mann–Whitney statistical test was applied to ascertain significance between groups. A significance level below 0.05 was considered statistically significant.

## 3. Results

### 3.1. Time Course of Disease Progression

**Figure 2:**
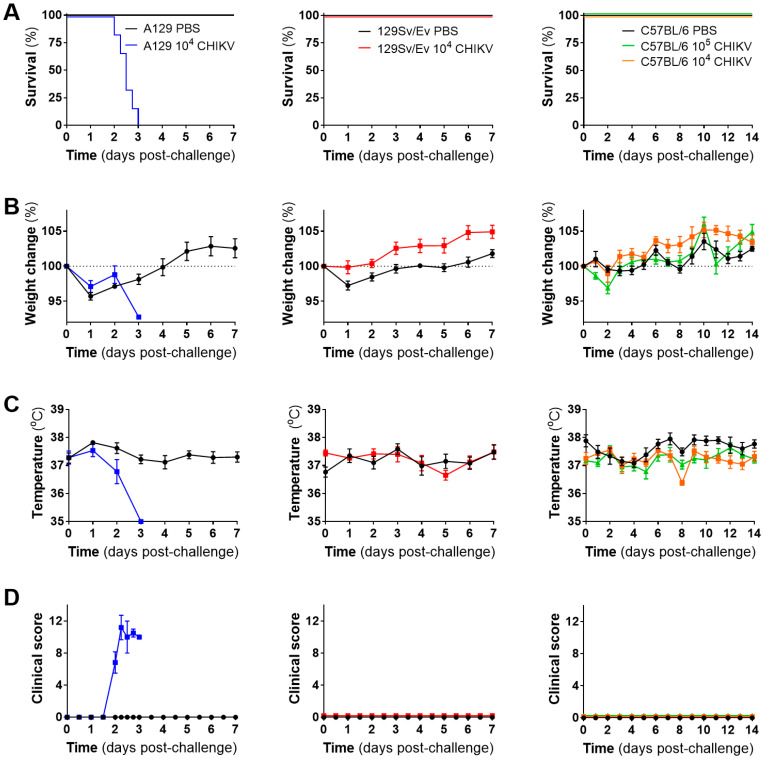
Clinical outcomes of mice challenged with CHIKV. (**A**) Kaplan–Meier survival plots, (**B**) weight changes compared to the day of challenge, (**C**) body temperature and (**D**) clinical scores. Lines show mean values with error bars denoting standard error. Results are shown from n = 6 animals per group pre-allocated for monitoring of clinical course for up to 7 days (A129 and 129Sv/Ev) or 14 days (C57BL/6) post-challenge.

CHIKV uniformly caused severe disease in strain A129 mice lacking their type-I IFN receptor, with all challenged animals meeting humane endpoints by day 3, whereas wild-type mouse strains all survived until the scheduled end of the study (Figure 2A). In most groups, weights decreased at the start of the study irrespective of whether challenged with CHIKV or the PBS control, likely as a result of moving into different accommodation involving flexible film isolators required for biological containment. Weight soon after increased across groups, with the exception of a single A129 mouse which met humane clinical endpoint on day 3 and had lost 7.25% weight since the day of challenge (Figure 2B). Temperatures in A129 mice decreased post-challenge compared to the PBS group, a trend not observed with the 129Sv/Ev or C57BL/6 mouse strains (Figure 2C). Clinical signs of disease were observed only in the CHIKV-challenged A129 mice, consisting of ruffled fur, arched back, lethargy, eyes closed and laboured breathing (Figure 2D). 

### 3.2. Viral Loads Measured in the Blood, Spleen, Limb and Brain at Day 3, 7 and 14 Post-Challenge

**Figure 3:**
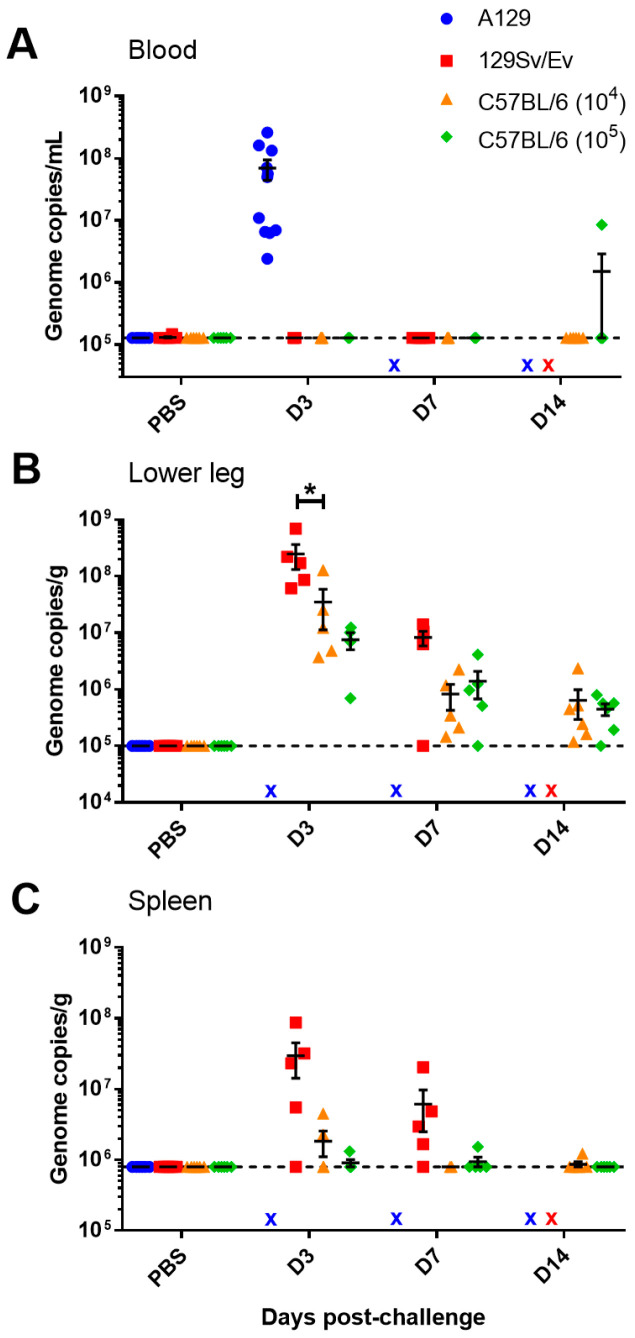
Viral RNA levels in blood and tissues from CHIKV-challenged mice measured by RT-PCR. Levels in the (**A**) blood expressed as genome copies per mL, and levels in the (**B**) lower hindlimb and (**C**) spleen expressed as genome copies per g. x, indicates samples not available. *, *p* < 0.05. Symbols show results from individual animals with line and whisker plots denoting mean and standard error.

To ascertain the tropism and dissemination of CHIKV during infection, animals from each challenge group were scheduled for cull on days 3 and 7 post-challenge to assess tissue responses. Due to all A129 mice reaching humane clinical endpoints by day 3 post-challenge, samples were not available at later timepoints. Also, due to reaching humane endpoints and the sudden deterioration in health, only blood was collected from these animals for viral load analysis. All A129 mice had high levels of CHIKV RNA in their circulation, unlike in the wild-type mice with the exception of a single C57BL/6 animal at the day 14 timepoint (Figure 3A). The main site of viral RNA was in the lower limb, close to where the viral inoculum was administered (Figure 3B). The levels of viral RNA in the lower limb were significantly higher in 129Sv/Ev mice compared to C57BL/6 mice given the same challenge dose at day 3 post-challenge. Similarly, viral RNA was also observed in the spleen in the majority of 129Sv/Ev mice, whereas only sporadic and lower levels were detected in the C57BL/6 strain (Figure 3C) although these differences did not reach the significance threshold (*p* = 0.0758 and *p* = 0.0601 for days 3 and 7 post-challenge, respectively). For all samples tested, there were no statistically significant differences between the 10^4^ and 10^5^ challenge doses in the C57BL/6 mouse strain. No PCR responses were observed in the brain. 

### 3.3. Pathological Findings

**Figure 4:**
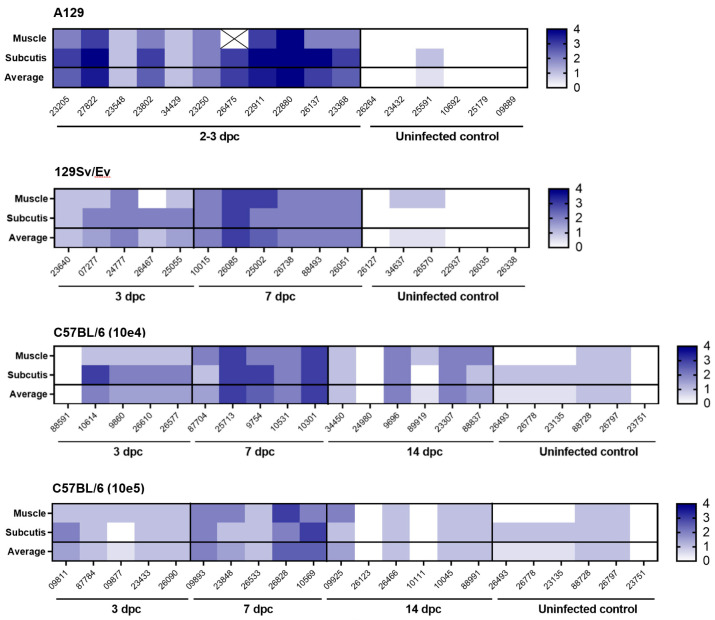
Heatmap illustrating the severity of microscopic changes in muscle and subcutaneous tissue (subcutis) of the hindlimb in individual animals from mouse strains A129, 129SvEv and C57BL/6. Values indicate individual and average severity scores (muscle and subcutis). Cross symbol indicates sample not available.

**Figure 5:**
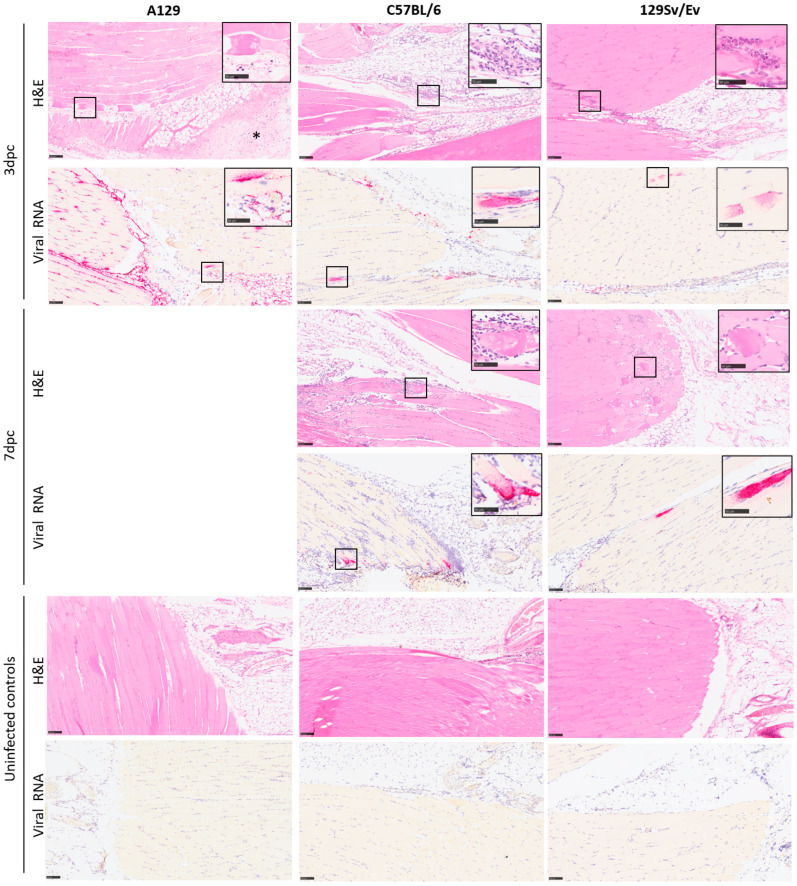
Representative images illustrating the type and severity of microscopic changes, and the presence of viral RNA staining, in the skeletal muscle and subcutis of the hindlimb from A129, C57BL/6 and 129Sv/Ev mouse strains at 3 and 7 days after challenge with 10^4^ pfu CHIKV, alongside uninfected controls. The changes comprise skeletal myocyte degeneration and loss, variably associated with a mainly neutrophilic cell infiltration and inflammation of the subcutis with oedema and haemorrhage and concomitant infiltrating inflammatory cells (indicated by an asterisk). These changes are more apparent in the A129 mice at 3 days post-challenge (left column, row 1) compared to C57BL/6 and 129Sv/Ev mouse strains at the same timepoint (middle and right columns, row 1). Viral staining is noted in all three strains (left, middle and right columns, row 2), and most prominent in A129 mice (left column, row 2). At 7 days post-challenge, there is a comparable increase in severity of changes in the C57BL/6 and 129Sv/Ev mouse strains (middle and right columns, rows 3 and 4) and low-level viral staining (middle and right columns, rows 4 and 5). Microscopic and changes and viral staining are absent in the uninfected control animals (left, middle and right columns, rows 5 and 6). Inset, higher power images of changes are highlighted in square boxes. Scale bars represent 100 μm in the main images and 50 μm in the insets. H&E, ISH.

Microscopic changes associated with infection with the CHIK virus was observed in a proportion of animals from each of the challenged groups at the various timepoints (Figure 4 and Figure 5). In the skeletal muscle, individual or groups of myocytes were observed to be degenerate or necrotic, with irregular-shaped cellular outlines and absent cytoplasmic cross striations, floccular, hyper-eosinophilic cytoplasm and nuclear karyorrhexis (coagulation necrosis). Variable numbers of mononuclear inflammatory cells, primarily macrophages, infiltrated the cells or were within the epimysium. In other areas, inflammatory cells infiltrated the endo- and peri- and epimysium of intact myocytes. Myocyte regeneration was also observed variably, comprising reduced diameter fibres with multiple, centralised nuclei and basophilic cytoplasm. Inflammatory cells extended into surrounding fibro-vascular and adipose connective tissue; this was variably associated with pale, eosinophilic material expanding collagenous tissue (oedema), vascular congestion and variable erythrocyte extravasation (haemorrhage). 

These changes were noted with the greatest severity in soft tissues associated with the tarsal joint of all A129 mice at days 2–3 post-challenge. In the other groups at the day 3 timepoint, changes were present in five/five animals in the 129Sv/Ev group, four/five animals in the C57BL/6 group challenged with 10^4^ pfu CHIKV, and five/five animals of the same strain challenged with 10^5^ pfu CHIKV. Appreciable differences in the severity of these changes between these three groups were not prominent. 

By contrast, by day 7 post-challenge, there was a trend towards increased severity of changes compared to the previous timepoint, the extent of which appeared comparable between the three groups. Only the C57BL/6 strain animals were examined at 14 days post-challenge; overall, the severity of disease had reduced and were similar between the two dosing regimens. Minimal changes were noted infrequently in the control groups that received a PBS inoculation.

Microscopic changes consistent with infection with CHIKV were not observed in either the spleen or brain of any animal in any group.

**Figure 6:**
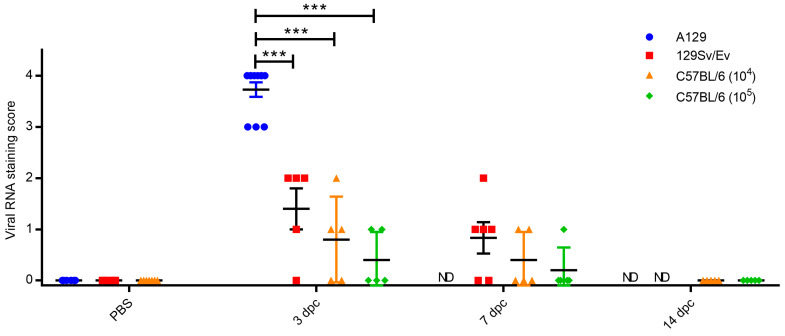
Levels of RNAscope staining in the muscle and subcutis of the hindlimb. PBS group are unchallenged animals whereas other groups were challenged with CHIKV and culled at 3, 7 and 14 days post-challenge (dpc). ND, not done due to animals meeting humane endpoints beforehand (A129) or not planned as part of the study schedule. Symbols show results from individual animals with line and whisker plots denoting mean and standard error. ***, *p* < 0.001.

Staining for CHIK viral RNA was present variably in a proportion of animals where microscopic changes had been identified (Figure 6). As expected, the degree of staining was greatest in tissues of animals in the A129 group at 2–3 days post-challenge, with severity scores ranging from 3 to 4 in all animals. At this timepoint, staining was also noted in four/five animals in the 129Sv/Ev group (severity scores 1–2); in three/five C57BL/6 animals that received the 10^4^ dose (severity scores 1–2); and in two/five animals that received the 10^5^ dose (severity scores 1–2). By day 7 post-challenge, positive staining was present in four/six 129Sv/Ev strain animals (severity scores 1–2), two/five C57BL/6 animals that received the 10^4^ dose (severity score 1); and in one/five animals that received the 10^5^ dose (severity score 1). In the C57BL/6 strain groups at 14 days post-challenge, viral staining was absent in all animals at both dose groups. Staining for viral RNA was absent in all control groups.

### 3.4. Cytokine, Chemokine and Growth Factor Levels Associated with CHIKV Infection

**Figure 7:**
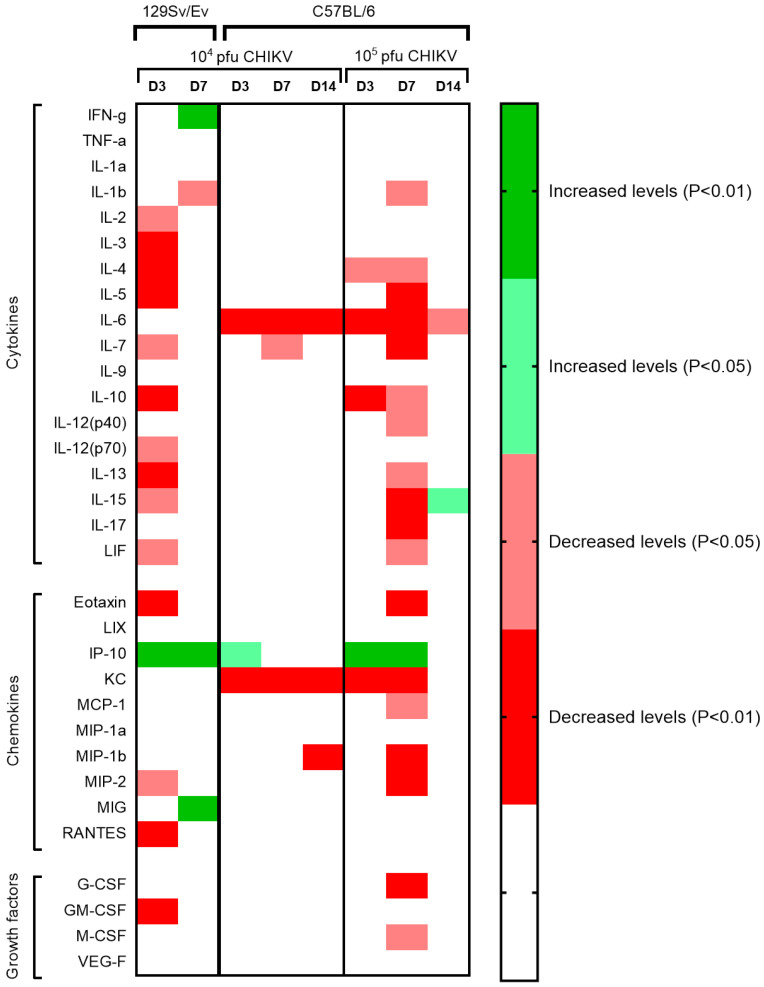
Heatmap showing statistically significant changes in cytokine, chemokine and growth factor serum concentrations in CHIKV-challenged animals compared to PBS mock-challenged strain-matched control animals (n = 6). Uncoloured areas represent no significance observed (*p* > 0.05).

Concentrations of 32 analytes were measured in the sera of animals challenged with CHIKV and compared with levels of those of the PBS control group. Statistical analysis showed that the majority of levels were significantly decreased during the acute phases of infection, with only levels of IFN-γ, IP-10 and MIG showing increased levels (Figure 7). The peak changes for the 129Sv/Ev mice were observed at 3 days post-challenge, whereas for the C57BL/6 strain it was at 7 days, with the latter showing a greater number of analytes affected at this timepoint with the higher challenge group. 

**Figure 8:**
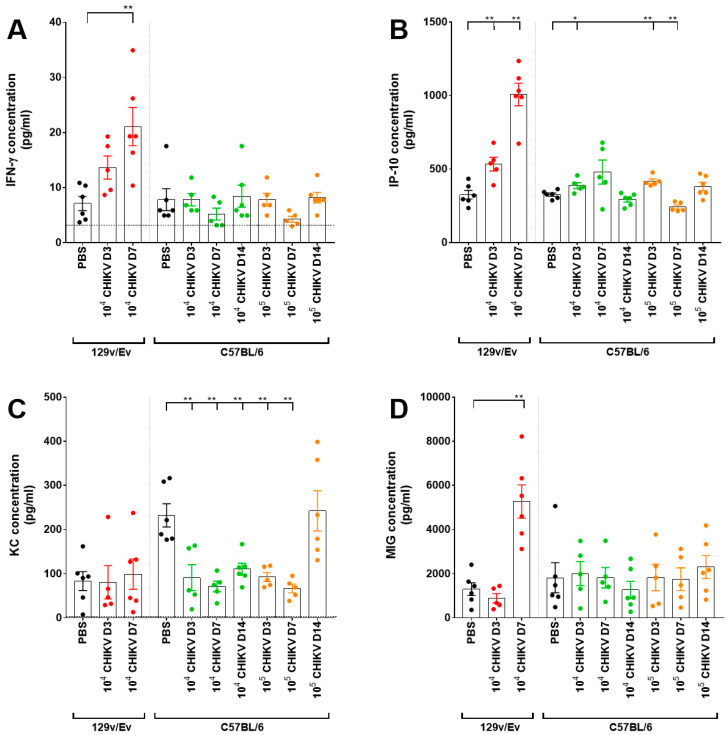
Concentrations of a subset of analytes demonstrating significant differences after challenge with CHIKV. Results show levels of (**A**) IFN-γ, (**B**) IP-10, (**C**) KC and (**D**) MIG. Bars represent the mean values with error bars denoting standard error and results from individual animals identified as coloured dots. *, *p* < 0.05; **, *p* < 0.01.

One cytokine (IFN-γ) and three chemokines (IP-10, KC and MIG) showing significant differences were further analysed. Levels of IFN-γ increased in 129Sv/Ev mice, reaching significance at the day 7 timepoint, in contrast to stable levels observed in C57BL/6 mice (Figure 8A). IP-10 levels were again clearly increasing in 129Sv/Ev mice, but with the C57BL/6 mice, although statistically significant differences were detected, the increases in levels were small (Figure 8B). The baseline levels of KC were higher in C57BL/6 mice compared with the 129Sv/Ev strain, with significant decreases observed in the former (Figure 8C). By day 14, KC levels had returned to normal in those challenged with 10^5^, but still remained subdued in those challenged with 10^4^. For MIG, the levels in all 129Sv/Ev-challenged mice at day 7 were higher than in the PBS group, with levels remaining consistent amongst other groups (Figure 8D). 

## 4. Discussion

Our results provide a direct comparison of CHIKV pathogenesis by applying identical study protocols in three mouse strains that are widely used for modelling arbovirus infection. The CHIKV strain used, LR 2006-OPY1, was isolated from a traveller returning from La Réunion in 2006 [30,31], where during this outbreak, over 260,000 cases (approximately one-third of the population) were reported [32]. This strain has also been widely used in challenge studies of non-human primates [33,34,35,36,37], thus our studies provide a direct comparison between these different preclinical model systems.

Direct comparisons were made between the two wild-type mouse strains, C57BL/6 and 129Sv/Ev. The results presented corroborate findings that C57BL/6 mice are a robust model for studying the pathogenesis of CHIKV-induced musculoskeletal disease [19]. Interestingly, in 129Sv/Ev mice, the levels of viral RNA in the lower leg and spleen were higher compared with the C57BL/6 strain, yet there was no evidence of viremia. The finding of viral RNA in the spleen indicates dissemination, and evidence of viral material 7 days post-challenge contrasts with reports that virus in this strain of mouse is cleared within 5 days [38]. However, our assay relied on the RT-PCR detection of viral RNA which may be more sensitive than live virus assays but has the limitation of not being able to distinguish between a viable virus and non-viable genetic nucleic acid. Our results provide evidence that 129Sv/Ev mice are a useful alternative to C57BL/6 mice, with increased and more prolonged virus in local tissues. In another comparison with CHIKV infection in wild-type mouse strains, CD-1 mice have also been shown to be comparable with the C57BL/6 strain [39].

Alongside the wild-type mouse strains, type-I IFN receptor-deficient mice were also assessed. All A129 mice met humane endpoints by day 3, slightly earlier than another study where survival dropped from day 4 [38]. This may be due to interpretation of endpoints, where in our study, humane clinical endpoints were set before initiation of the study whereas in the latter, the study was reported as a morbidity/mortality design. The advantage of A129 mice is the ability to measure survival as a clear outcome post-challenge, as has been demonstrated with CHIKV vaccines based on chimpanzee adenovirus vectored constructs [40,41]. The A129 mice showed consistent viremia compared with the wild-type mouse strains, as seen in humans and at similar levels to the 7 log_10_ magnitude reported [42]. Due to a rapid deterioration in health and multiple animals reaching humane endpoint simultaneously, tissues were not able to be sampled at necropsy. The finding of viremia demonstrates further utility of this strain to be used for vector competence studies, as reported for the similar AG129 mice with an additional knockout for their IFN-γ receptor [43].

Across the different mouse strains, a consistent challenge dose of 10^4^ pfu was delivered to enable comparability. This dose was used in an assessment of different CHIKV strains, including LR 2006 OPY1 used in our study, to assess lineage-specific differences in virulence using the A129 mouse model after footpad inoculation with all strains resulting in animals meeting humane endpoints [44]. In other reports using the same CHIKV-challenge strain as used herein, a vaccine study using Balb/C mice similarly challenged with 10^4^ pfu by the subcutaneous (s.c.) route [45]. In C57BL/6 mice, 10^4^ pfu was also used, but with the s.c site being at the base of the tail [46]. Higher doses of 10^6^ TCID_50_ in C57BL/6 mice have been reported, resulting in a transient and recoverable disease progression [47]. Whilst TCID_50_ titres are not directly comparable to pfu, an efficacy study in C57BL/6 mice used a challenge dose of 10^5^ pfu via the s.c. footpad route [48]. Infectious clones of LR 2006 OPY1 were used to develop a challenge model in C57BL/6 mice, with doses of 10^4^, 10^5^ and 10^6^ pfu all demonstrating similar levels of viraemia with no discernible differences observed [49]. Given these higher doses of CHIKV strain LR 2006 OPY1 in C57BL/6 mice, this provided the reasoning for inclusion of an additional group of 10^5^ pfu in this mouse strain. 

Foot swelling has been observed with CHIKV infection in mice but was not observed in the studies described herein. Some studies have been conducted in younger C57BL/6 mice, such as those 14 days old [19], whereas others were in mice more aligned with being over 6 weeks old [18,50]. Our findings of failing to detect swelling aligns with previous reports conducted with wild-type 129Sv/Ev mice where no swelling of the contralateral hind footpad was detectable [38]. In C57BL/6J mice, there was no change in limb thickness in animals challenged with 10^2^ and 10^4^ pfu, but it was increased with a higher dose of 10^6^ pfu using an Indian CHIKV isolate [51]. After inoculation at the base of the tail, foot swelling was also absent despite the presence of viraemia, alongside the finding that non-injected feet do not demonstrate clinical effects [18]. Therefore, the lack of swelling may be due to minor differences in the injection sites, as the foot/footpad is used in many reports [18,19], but due to welfare considerations and discomfort of the animals post-inoculation, a site slightly above the foot was used in our studies. This argument is reinforced by others, who found that delivery of CHIKV via the flank of C57BL/6 mice did not result in detectable foot swelling, but when delivered to the feet, swelling was observed [49]. Mosquito-mediated human infection results in swelling across multiple joints [52], and not just those directly where the biting occurred, thus presenting a limitation of the C57BL/6 mouse model as these effects are not recapitulated. Interestingly, when this was tested in BALB/c mice, after inoculation on the back, swelling and inflammation of the legs was observed [53], indicating differences between mouse strains but this was more likely due to the young age (2–3 days) of the mice used which are more susceptible to alphaviral disease [54].

The type of histopathological lesions observed in the skeletal muscle and subcutis associated with the tarsal joint of the C57BL/6 strain of mice are similar with published data [18,19,50,55]; unfortunately, due to limitations in the processing of tissues, the joints and bone were not included in the evaluation. Similar types of pathological changes were noted in the other two mouse strains with varying severity, with the most severe pathology, as predicted, noted in the A129, type-I IFN receptor-deficient mice. The severity of changes observed in the three mice strains at the varying timepoints were consistent with the changes seen in the viral and clinical data.

At set timepoints post-challenge, planned culls were scheduled to ascertain local responses at days 3 and 7 post-challenge based on evidence from published studies. Day 3 was chosen due to viremia at days 2 and 3 being higher than day 1 in A129 mice [44,45]. In addition, days 3 and 7 also correlated with the biphasic disease progression in the wild-type C57BL/6 mice, where peak disease/morbidity scores were observed on day 3 in two independent studies, and then another at day 7 or soon after [47,50]. With the aim of our work being to compare the different mouse strains (A129, 129Sv/Ev and C57BL/6), we chose representative timepoints relevant to these. For further interrogation of the A129 mouse strain, due to the rapid progression of disease, earlier timepoints specific to this strain are warranted and for the wild-type strains, and additional later timepoints should be considered.

During early infection events, levels of the chemokine IP-10 were consistently increased in CHIKV-challenged 129Sv/Ev and C57BL/6 mice. MIG and IFN-γ were also elevated, but only in 129Sv/Ev mice. These align with studies in acute CHIKV patients. In a study of 196 acute patients across tertiary care hospitals across India, levels of IP-10, IFN-γ and MIG sera levels correlated with disease severity [56], although a fourth marker which was also found to be significant, MCP-1, did not show correlation in our challenged mice. In 88 CHIKV patients in Brazil, levels of circulating IP-10 and IFN-γ were increased during the acute phase [57], further aligning with our findings in CHIKV-challenged mice; although other markers were also increased in the patients such as TNF-α, IL-7 and MCP-1 which were not significant in our mouse models. Another study in Brazil comparing 29 acute CHIKV patients with 21 healthy controls also found significantly increased levels of IP-10, MIG and IFN-γ in the patient sera, alongside IFN-α, IL-6, IL-8, IL-10 and MCP-1 [58]. Similarly, in acutely infected CHIKV patients, levels of IP-10 were increased after studying levels from 69 patient from the Gabonese outbreak of 2007, but again, alongside several other analytes [59]. IFN-γ has been shown to be elevated in similar studies in C57BL/6 mice, but alongside other biomarkers including TNF-α, MCP-1, IL-6 and IFN-α/β [18].

The majority of other cytokines were either substantially reduced or unchanged compared with control groups. A meta-analysis of biomarkers in CHIKV infection found that decreased levels of RANTES and IL-8 had an association with disease severity; however, the rise of a significant number of analytes (including those included in the mouse panel: IL-6, TNF-α, MCP-1, IL-12, IL-13, GM-CSF, IL-1 α, IL-15, IL-10, IL-4, MIP-1α, and MIP-1 β) were associated with chronification [60]. The levels in mice being decreased for many of these analytes suggests that the infection in these mice is resolving instead of progressing to a chronic state. The finding of fewer analytes being raised in our studies may arise from differences in levels of sensitivities between different assays used or degradation of cytokines, due to their intrinsically short half-life in blood [61]. Due to decontamination and logistical processes from collection at necropsy to processing the sera in an in vitro laboratory and frozen storage, delays may have resulted in the deterioration of the quality of the sample. However, in previous work on Zika virus, a similar approach was undertaken with a wider range of biomarkers showing elevated levels [62], thus this is unlikely an effect due to the condition of the sera being tested. 

In summary, our results point towards two separate CHIKV-induced disease sequelae in mice which should be measured in studies on the impact of vaccines and therapeutics. The A129 mouse model, for which CHIKV produces a uniformly lethal disease, allows survival readouts as a relevant endpoint for success criteria of intervention testing. Of the wild-type mice strains tested, in the absence of clinical disease, viral loads and histological changes would be appropriate readouts. In that regard, viral loads were higher in 129Sv/Ev compared with C57BL/6 in key sites; thus, the former strain may prove more sensitive when incomplete protection is conferred by the intervention and would be preferable for deciphering partial control of virus replication and other protective effects. 

Given the lack of feasibility to conduct efficacy studies due to the unpredictable nature of CHIKV outbreaks and incomplete epidemiology, in part due to inadequate surveillance [63], animal models form a key component for vaccine licensure. The use of immunogenicity endpoints as a surrogate of protection, based on the passive transfer of human post-vaccination sera to a susceptible animal model (e.g., NHP) prior to wild-type challenge to ascertain protective effects [64] in the licensure of the first human CHIKV vaccine, demonstrates the value of in vivo models which resemble the spectrum of human disease manifestations. 

## Data Availability

The data presented in this study are available upon request from the corresponding author.

## Appendix A. Scoring Criteria for the Subjective Assessment of Microscopic Changes in Hindlimb Soft Tissues Resulting from Infection with Chikungunya Virus in Mice

**Table:**

Lesion	Score				
	0 (Normal)	1 (Minimal)	2 (Mild)	3 (Moderate)	4 (Marked)
Skeletal muscle					
Skeletal myocyte degeneration/necrosis ± inflammatory cell infiltration, mainly neutrophils.	None	Occasional, scattered single or small groups of degenerating myocytes—up to 5% of skeletal muscle affected.	Increased numbers of degenerating myocytes—6–25% of skeletal muscle affected.	Frequent numbers of degenerating myocytes—26–50% of skeletal muscle affected.	Numerous degenerating myocytes—>50% of skeletal muscle affected.
Skin and subcutis					
Vascular congestion, oedema and haemorrhage with a mixed inflammatory cell infiltrate, often predominantly neutrophils, with fewer macrophages and lymphocytes.	None	Up to 5% of skin and soft connective tissues affected.	6–25% of skin and connective tissues affected.	26–50% of skin and connective tissues affected.	>50% of skin and connective tissues affected.
